# Gene copy number variation in Indian population and its implication in health

**DOI:** 10.1186/1755-8166-7-S1-P119

**Published:** 2014-01-21

**Authors:** Suhani Almal, Harish Padh

**Affiliations:** 1 Cellular and Molecular Biology, B. V. Patel Pharmaceutical Education & Research Development (PERD) Centre, Ahmedabad-380054, India

## Objectives

Copy number variations (CNV) are important source of human genetic variation, found to be widely prevalent than what was initially predicted. The involvement of CNVs in disease susceptibility and drug response is well reported in other populations but has not been studied to a larger extent in Indian population. The aim of the present study was to evaluate the distribution of copy number variable genes in Indian population and their link, if any, to health, disease and drug response along with development of a comprehensive resource of CNV frequency distribution among different populations.

## Methods

A total of 100 – 280 healthy controls and in variable number of patients from Indian population were genotyped. Genotyping of the copy number (CN) variable genes was carried by PCR-based methodologies (long range PCR, real-time PCR and PRT assay).

## Results

An indicative correlation with disease susceptibility and significant (p < 0.05) difference in the frequency distribution of the CNV variants was observed in our study. MTUS1 deletion variant was found to be significantly (p = 0.0207) associated with a decreased risk to breast cancer. A significant association between *CCL3L1* copy number and risk to HIV-1 was also observed. The inter-ethnic comparison of *CCL3L1* gene copy number frequency demonstrated significant difference worldwide, being highest in Africans. The common deletion frequency comprising *LCE3B* and *LCE3C* genes was found to be highest in European and American populations. The frequency of FCGR3B CN < 2 was found comparatively higher in Indians, Americans and Africans as compared to European Caucasians. Thus, inter-ethnic differences were observed among populations, highlighting varied frequency distribution pattern based on their distinct geographical location.

## Conclusion

This is our first attempt to create a comprehensive map of the frequency distribution of CNVs and its association to disease susceptibility in Indian population. This varied worldwide distribution can thus be relevant from clinical or evolutionary point of view in future.

